# Treatment adherence among sputum smear-positive pulmonary tuberculosis patients in mountainous areas in China

**DOI:** 10.1186/1472-6963-11-341

**Published:** 2011-12-16

**Authors:** Song Yao, Wen-Hui Huang, Susan van den Hof, Shu-Min Yang, Xiao-Lin Wang, Wei Chen, Xue-Hui Fang, Hai-Feng Pan

**Affiliations:** 1 Anhui Provincial TB Research Institute; Hefei, China; 2 Jiangxi Provincial Center for Disease Control and Prevention; Nanchang, China; 3 KNCV Tuberculosis Foundation, The Hague, the Netherlands; 4 Center for Infection and Immunity Amsterdam (CINIMA), University of Amsterdam, Amsterdam, The Netherlands; 5 Gansu Provincial Center for Disease Control and Prevention; Lanzhou, China; 6 Ningxia Provincial Center for Disease Control and Prevention; Yinchuan, China; 7 Guizhou Provincial Center for Disease Control and Prevention; Guiyang, China; 8 Department of Epidemiology and Biostatistics, School of Public Health, Anhui Medical University, Hefei, China

## Abstract

**Background:**

We carried out an investigation in five provinces in China to assess treatment adherence and identify factors associated with insufficient treatment adherence in tuberculosis (TB) patients in mountainous, rural areas of China.

**Methods:**

In each of the five provinces, all counties with > 80% mountainous area were stratified into three groups according to their gross domestic product. In each stratum, one county was randomly sampled. Study subjects were sampled from all smear positive TB cases registered in 2007 in the target counties. TB patients, village doctors, county doctors and directors of the TB prevention and control institutes were interviewed. Insufficient medication adherence was defined as taking less than 90% of anti-TB drug doses prescribed. Insufficient re-examination adherence was defined as having less than the recommended three sputum smear examinations during the treatment course.

**Results:**

A minority of patients took drugs under direct observation: on average 29% during the intensive phase of treatment. In total, 524 TB patients were included, of whom 49 (9.4%) took less than 90% of all doses prescribed and 92 (17.6%) did not have all sputum smear examinations, with substantial variations between the provinces. In multivariable analysis, no direct observation of treatment during the intensive phase and the presence of adverse events were associated both with insufficient medication adherence and insufficient re-examination adherence. Overall, 79% of patients were adherent both to treatment and re-examinations.

**Conclusions:**

In these remote and poor areas of China, the TB control program is not fully functioning according to the guidelines. The majority of patients are not treated under direct observation, while direct observation by health care staff was associated with better adherence, both to drug therapy and re-examinations. Insufficient adherence increases the risk of unsuccessful treatment outcomes and development of drug resistance. Measures should be taken urgently in these areas to strengthen implementation of the international Stop TB strategy.

## Background

Adherence, or compliance, refers to the extent patients correctly follow medical advice [1]. Adherence refers both to taking drugs according to prescription and to adherence to other recommendations such as attending medical check-ups. Within the international tuberculosis (TB) control strategy [2], direct observation of treatment (DOT) was introduced to improve treatment outcomes [3]. Non-adherence increases the risk of prolonged infectiousness, development of drug resistance, relapse of TB, and death [3-10]. Barriers to adherence include poor literacy of patients on health in general and lack of comprehension of benefits of treatment and risks of prematurely stopping treatment specifically, occurrence of side effects, and costs [11].

China ranks second on the list of countries with the highest number of yearly, incident TB cases [12]. In 2009, an estimated 1.3 million individuals in China got TB. A recent study in Jiangsu province in China found that 12% of patients did not take at least 10% of their prescribed doses of anti-TB treatment [13]. Another study in Hebei province however observed that 46% missed at least 10% of the doses [14]. Several other studies in China also indicated low treatment adherence among sputum smear-positive pulmonary tuberculosis patients [15-18]. However, only one province or one city had been investigated in these previous studies. Moreover, studies in mountainous areas of China are very limited. In rural, mountainous areas in China, limited access to transportation, the large service area of health clinics and village doctors, and poverty may complicate access to health care for diagnosis and treatment of TB even more than in other rural areas [19]. We carried out an investigation in five provinces in China to assess treatment adherence and identify factors associated with insufficient treatment adherence in tuberculosis patients in mountainous, rural areas of China. These results may be used to develop targeted measures to improve patient treatment adherence and hereby improve cure and reduce development of drug resistance and recurrence of TB.

## Methods

### Study population and sampling

This cross-sectional comparative study was conducted in counties with at least 80% of the population living in mountainous areas. Five provinces with a significant proportion of mountainous area in their province participated in the study: Anhui, Ningxia, Gansu, Jiangxi and Guizhou. Situated inland astride the Yangzi (Yangtse or Changjiang) River, Anhui is a rich agricultural land on the fringes of the North China plain. Situated near the geographical central northern edge, Ningxia Province belongs more naturally to the North-Western group of provinces. Gansu Province is the north-western corridor into China, bounded by inhospitable, mountainous Qinghai to the south-west and the Gobi desert to the north-east, it forms the natural route for the overland silk routes into the heartland of China. Jiangxi is a fairly mountainous inland province with a long history. The town of Jingdezhen is famous for its blue and white porcelain. Guizhou is not one thing or another, it's near the border with Vietnam but not on the border and in the mountains but not the high mountains, it has a somewhat transitional climate too, between the tropical and the continental types.

Of all counties in these provinces, 17 (16%), 8 (36%), 46 (53%), 42 (42%), 75 (85%) respectively, had at least 80% of the population living in mountainous areas. Per province, these mountainous counties were stratified in three strata (tertiles) according to the provincial per capita gross domestic product (GDP). Per stratum, one county was randomly selected so that in total 15 counties, three from each of five provinces, were included in the sample.

We calculated that with a sample size of 500, assuming a rate of exposed to unexposed of 1:2 and 25% chance of disease among the unexposed, we would be able to demonstrate an odds ratio of minimally 2 with alpha = 0.05 and beta = 0.10. Therefore, we aimed to interview 35 smear-positive pulmonary TB patients per county.

We randomly sampled smear positive cases from all smear positive cases registered in 2007 in the target mountainous counties. Sampling was performed in batches until 35 cases per county had been included. In one county in Ningxia province, 34 cases were included. A sample of 556 individuals was necessary to include these 524 (94%) patients. The main reason for non-participation was individuals having migrated out of the county for work.

### Data collection

We examined which patient related factors (e.g. age and sex), healthcare provider related factors (e.g. treatment supervision), and socio-economic factors (e.g. local GDP) were associated with poor adherence to anti-TB drugs and poor adherence to smear examinations during the treatment period.

A total of 524 TB patients were interviewed at the county level TB institutes by county level TB staff, using structured questionnaires on treatment adherence and factors associated with low treatment adherence. Each item of the questionnaire was carefully explained to the study subjects, all the questionnaire was administered by trained interviewers. A preliminary investigation was carried out before formal study.

In order to assess which reasons TB physicians thought to be associated with insufficient adherence, structured interviews were held with the director of each county TB dispensary, with doctors at the county level TB dispensary, and with doctors at the township and village level. TB dispensary doctors prescribe treatment and examine patients during follow-up medical examinations while township and village doctors monitor treatment.

The same protocol was used in each province. In each province, county level investigators were trained by the provincial level investigators during a one day workshop before data collection. Data collection took place between October 2008 and January 2009, so that the whole 2007 cohort had finished treatment.

In the standardized thrice-weekly treatment regimen new patients should take drugs 90 times and smear-positive retreatment patients 120 times. If there are one or more sputum smear-positive results during treatment, then treatment will be extended. The total number of times the patients should have taken drugs were taken from the patient medication record card. A patient was defined to have good adherence to treatment if the proportion of actual doses taken of those prescribed was at least 90%.

According to the national TB program (NTP) guideline, both new and retreatment patients should have three sputum smear examinations during the treatment course (at 2, 5, 6 after the start of treatment for new patients and 2, 5, 8 months for retreatment patients). A patient was defined to have good adherence to smear re-examinations if he or she had at least three smear re-examinations.

After completion of questionnaire investigation by the county level staff, provincial research staff re-interviewed 10% of included patients (4 per county). In all counties, re-interviews gave similar results.

### Data analysis

Data were double entered using EpiData version 3.1, and all statistic analyses were performed by Statistical Package for Social Sciences (SPSS), version 10.0. Chi-square tests were used to identify associations between patient and treatment characteristics and treatment adherence. Separate analyses were performed for risk factors for insufficient adherence to taking of anti-TB drugs, and for insufficient adherence to sputum smear re-examinations. To assess independent risk factors for non-adherence, variables with a P-value < 0.2 in univariate analysis were included in multivariable logistic regression models. By backward selection based on the -2 log likelihood ratio, the final model was selected. The level of significance was set at *P *< 0.05.

### Ethical considerations

The investigation was carried out after written informed consent of study participants. The study was approved by the Ethics Committee of the national TB control association.

## Results

### Study population characteristics

In the study population consisting of 524 TB patients, the average age was 47.7 (standard deviation 17.5) years, 364 (69.5%) were males, and 427 (81.5%) were farmer. Overall, 142 (27.1%) of the patients had no formal education while a further 172 (32.8%) had no education beyond primary school. The distribution of demographic characteristics of the TB patients and characteristics of their treatment are shown in more detail in Table 1. Between provinces, striking differences were observed in the proportion of patients without formal education, the proportion of patients that were aware of the free policy for TB diagnosis and treatment, the distances from patients' homes to village clinics, the proportion of patients receiving DOT during the intensive phase, and the proportion of patients receiving DOT during the continuation phase (P-values < 0.001). The proportion of patients without formal education ranged between 8.6% and 54.8%, and the proportion of patients that were aware of the free policy for TB between 44.8% and 89.5%. On average, 21.8% lived more than 2 kilometers from the village clinic and 30.9% lived more than 40 kilometers from the re-examination center. Despite differences, the proportion of patients receiving DOT was low in all provinces: it ranged between 9.5% and 43.3% during the intensive phase and between 8.6% and 37.5% in the continuation phase.

**Table 1:** Description of TB patients and their treatment per province

	Anhui (n = 105)	Ningxia (n = 104)	Jiangxi (n = 105)	Guizhou (n = 105)	Ganshu (n = 105)	Total (n = 524)
**participation (%)**	95.2	94.2	93.3	92.4	94.3	94.2

**male (%)**	77.1	59.6	78.1	69.5	62.9	69.5

**Mean age (SD) in years**	51.5 ± 16.4	46.6 ± 17.7	44.7 ± 13.9	46.4 ± 18.8	49.1 ± 19.5	47.7 ± 17.5

**previously treated patients (%)**	2.9	15.4	7.6	10.5	9.5	9.2

**without formal education (%)**	17.1	54.8	8.6	16.2	39.0	27.1

**aware of free policy for TB diagnosis and treatment (%)**	49.5	70.2	74.3	44.8	89.5	65.6

**Distance from home to village clinics (%)**						

**< 1 km**	42.9	37.5	29.5	5.7	53.3	33.8

**1-2 km**	40.0	18.3	47.6	55.2	29.5	38.2

**> 2 km**	17.1	44.2	22.9	39.1	17.2	28.1

**Distance from home to diagnostic and re-examination center**						

**< 20 km**	36.2	30.8	43.8	21.9	39	34.4

**20-40 km**	27.6	34.6	40	35.2	36.2	34.7

**> 40 km**	36.2	34.6	16.2	42.9	24.8	30.9

**with DOT during intensive phase (%)**	26.7	43.3	31.4	9.5	35.2	29.2

**with DOT during continuation phase (%)**	24.8	37.5	30.5	8.6	28.6	26.0

SD = standard deviation; DOT = direct observation of treatment

### Adherence to taking of anti-TB drugs

On average, forty-nine (9.4%) of patients reported having taken less than 90% of the drug doses prescribed. This percentage ranged from 0% in Ningxia province to 10.5% in Anhui province (p = 0.02) (Table 2). Characteristics associated with poor adherence in univariate analysis were province, not having a formal education, no supervision visits by (village) doctor during treatment, adverse effects during treatment, no DOT during the intensive phase, no DOT during the continuation phase, and DOT conducted outside the patient's home or no DOT conducted as compared to home-based DOT (Table 2). In multivariate analysis, independent risk factors for poor adherence were experiencing adverse effects during treatment, no DOT during the intensive phase and no home-based DOT. Observation of treatment by volunteers tended also to decrease the chance of non-adherence (p = 0.06).

**Table 2:** Tuberculosis patient and treatment characteristics associated with taking less than 90% of prescribed doses of anti-TB treatment

		< 90% drug adherence		Univariate			Adjusted	
	**N**	**n**	**%**	**OR**	**95% CI**	**p-value**	**OR**	**95% CI**

**Province**						0.02		

Anhui	105	11	10.5%	1.00				

Ningxia	104	0	0%	--	--			

Jiangxi	105	9	8.6%	0.80	0.32-2.02			

Guizhou	105	22	21.0%	2.27	1.04-4.95			

Ganshu	105	7	6.7%	0.61	0.23-1.64			

**Education level**						0.04		

No formal education	142	7	4.9%	1.00				

At least primary school	382	42	11.0%	2.38	1.04-5.43			

**TB patient category**						0.20		

New	476	42	8.8%	1.00				

Retreatment	48	7	14.6%	1.76	0.75-4.18			

**Adverse effects during treatment**						< 0.01		

Yes	312	41	13.1%	1.00			1.00	

No	212	8	3.8%	0.26	0.12-0.57		0.30	0.17-0.53

**DOT during the intensive phase**						< 0.01		

no DOT	241	33	13.7%	1.00			1.00	

DOT by village doctors	153	5	3.3%	0.21	0.08-0.56		0.28	0.09-0.82

DOT by volunteers	130	11	8.5%	0.58	0.28-1.20		0.44	0.19-1.03

**DOT during continuation phase**						0.02		

no DOT	253	31	12.3%	1.00				

DOT by village doctors	136	4	2.9%	0.22	0.08-0.63			

DOT by volunteers	135	14	10.4%	0.83	0.42-1.62			

**Supervision visits by (village) doctor during treatment**						< 0.001		

Yes	462	35	7.6%	1.00				

No	62	14	22.6%	3.56	1.79-7.08			

**Time DOT observer spends with the patient during observation of treatment**						0.003		

DOT at home, ≤10 minutes	181	10	5.5%	1.00			1.00	

DOT at home, > 10 minutes	198	15	7.6%	1.40	0.61-3.20		2.10	0.82-5.42

DOT outside home or no DOT	145	24	16.6%	3.39	1.57-7.35		3.49	1.42-8.62

### Adherence to sputum smear re-examinations

On average, ninety-two (17.6%) patients did not have all three re-examinations. This proportion ranged from 9.5% in Anhui province to 49.5% in Guizhou province (p < 0.001) (Table 3).

**Table 3:** Tuberculosis patient and treatment characteristics associated with incomplete attendance of re-examinations during treatment

		Incomplete re-examination		Univariate			Adjusted	
	**n**	**n**	**(%)**	**OR**	**(95% CI)**	**p-value**	**OR**	**(95% CI)**

**Province**						< 0.001		

Anhui	105	10	9.5%	1.00			1.00	

Ningxia	104	7	6.7%	0.69	0.25-1.88		0.83	0.29-2.3

Jiangxi	105	12	11.4%	1.23	0.51-2.98		1.30	0.53-3.2

Guizhou	105	52	49.5%	9.32	4.38-19.84		11.64	5.1-26.7

Ganshu	105	11	10.5%	1.11	0.45-2.74		2.24	0.84-5.9

**TB patient category**						0.16		

New	476	80	16.8%	1.00				

Retreatment	48	12	25%	1.65	0.82-3.31			

**Gender**						0.05		

male	364	72	19.8%	1.00				

female	160	20	12.5%	0.58	0.34-0.99			

**Age (years)**						0.12		

≤45	238	36	15.1%	1.00				

45 -65	193	33	17.1%	1.16	0.69-1.94			

> 65	93	23	24.7%	1.84	1.02-3.33			

**Awareness of free diagnosis and treatment policy**						0.19		

Yes	344	55	16.0%	1.00				

No	180	37	20.6%	1.36	0.86-2.16			

**Patients had heard about tuberculosis before seeking care**						0.10		

Yes	297	45	15.2%	1.00				

No	227	47	20.7%	1.46	0.93-2.30			

**Distance from home to village clinics**						0.11		

< 1 km	177	23	13%	1.00				

1-2 km	200	37	18.5%	1.52	0.86-2.67			

> 2 km	147	32	21.8%	1.86	1.04-3.35			

**Return trip costs to diagnostic and re-examination center**						0.008		

≤20 RMB	373	55	14.7%	1.00				

> 20 RMB	151	37	24.5%	1.88	1.18-3.00			

**Adverse effects during treatment**						< 0.001		

Yes	312	71	22.8%	1.00			1.00	

No	212	21	9.9%	0.37	0.22-0.63		0.33	0.18-0.59

**DOT during the intensive phase**						0.12		

no DOT	241	51	21.2%	1.00				

DOT by village or town doctors	153	24	15.7%	0.69	0.41-1.18			

DOT by volunteers	130	17	13.1%	0.56	0.31-1.02			

**DOT observer during ambulatory treatment**						0.006		

No DOT observer	138	44	31.90%	1.00			1.00	

Family member, volunteer or other	230	31	13.50%	0.33	0.20-0.56		0.61	0.29-1.3

village doctor or township doctor	156	17	10.90%	0.26	0.14-0.49		0.33	0.18-0.63

**Supervision visits by (village) doctor during treatment**						< 0.001		

Yes	462	69	14.9%	1.00				

No	62	23	37.1%	3.36	1.89-5.97			

**time DOT observer spends with the patient during observation of treatment**						0.006		

DOT at home, ≤10 minutes	181	31	17.1%	1.00				

DOT at home, > 10 minutes	198	24	12.1%	0.67	0.38-1.19			

DOT outside home	100	21	21.0%	1.35	0.76-2.63			

no DOT	45	16	35.6%	2.24	1.45-3.72			

In univariate analysis, factors associated with incomplete adherence to re-examinations were province, male gender, adverse effects during treatment, return trip costs to the diagnostic and re-examination center > 20 RMB, no supervision visits by (village) doctor during treatment, and not having a DOT observer during treatment at homeland (Table 3). Independent risk factors associated with incomplete adherence to re-examinations were province, experiencing adverse effects, and not having a DOT observer at home.

### Structured interviews with TB doctors

A total of 85 village doctors, 32 county doctors and 15 directors of county-level tuberculosis prevention and control institutions from five provinces were interviewed. TB doctors from the township/village and county level attributed insufficient treatment adherence to: adverse effects (50 doctors, 43%), difficulties associated with traffic and distances in mountainous areas (26 doctors, 22%), insufficient awareness of TB and insufficient attention to TB symptoms (16 doctors, 14%), migrant work (12 doctors, 10%), financial difficulties associated with TB disease and treatment (11 doctors, 10%) and the long duration of anti-TB treatment (9 doctors, 8%). The main difficulties village doctors encountered in TB work were a lack of government funding for TB control tasks (28 doctors, 32.9%), inconvenient traffic and distances in mountain areas (19 doctors, 22.4%), low treatment adherence of patients (10 doctors, 11.8%) low education level of patients (6 doctors, 7.1%), and high workload (5 doctors, 5.9%). The main difficulties county doctors encountered in their work were workplace related: lack of government funding (n = 10, 31%), outdated equipment (n = 7, 21.9%), and insufficient human resources (n = 6, 18.8%). In addition, low treatment adherence was mentioned relatively often as a difficulty encountered in their work (n = 7, 21.9%).

Of the 15 directors of the county-level TB prevention and control institutions interviewed, 5 considered government funding for TB control to be insufficient to meet operational needs.

## Discussion

In our study population from five provinces, on average 9.4% (range: 0%-21.0%) of TB patients missed more than 10% of the number of prescribed doses and on average 17.6% (range: 6.7%-49.5%) of them did not go to the CDC for all smear re-examinations. The proportion of non-adherent patients was lowest in Ningxia province and highest in Guizhou province. Overall, 79% of patients complied sufficiently both to treatment and re-examinations.

DOT aims to improve adherence in order to improve treatment outcomes and prevent creation of drug resistance. Our results show that DOT is not implemented well in the five mountainous provinces of China included in our study. During the intensive phase, on average 71.2% of TB patients were not treated under direct observation and this proportion ranged from 56.7% in Ningxia province to 90.5% in Guizhou province. Our results is similar to the results of Sun et al [15], they reported the majority of TB patients in rural areas do not receive DOT from village doctors and rarely get support, such as visits as required, from the CTBDs or township health providers in Shandong, China. Another study in Chongqing by Hu et al also found DOT is not well implemented, only less than 5% of TB patients (17/401) were observed by health staff [18].

The patients included from Guizhou province least often got treatment under direct observation and had significantly lower adherence to both medication and sputum re-examinations. Lack of treatment under direct observation and experiencing adverse events during treatment were the only independent risk factors which negatively influenced adherence both to treatment and to re-examinations in our study. Re-examinations during treatment are important to monitor the response to the therapy and allow identification and management of adverse events, which in turn may help to prevent non-adherence to therapy including default. A recent study from China showed that many patients were not aware of the importance of sputum re-examinations [20]. It has been observed before that adverse events during treatment increase the risk of non-adherence to therapy [13,20-25]. Driver [26] studied determinants for treatment interruption and found that the lack of TB disease and treatment related knowledge were important reasons for patients discontinuing treatment. Indeed, health education has shown to improve treatment adherence [24]. In addition, pscychosocial support from family, friends and professionals can improve patient adherence [24-28].

The doctors in fact mentioned that low treatment adherence was a problem they encountered in their work. A high workload and the remoteness of the areas they work in may prevent them to take appropriate actions to supervise patients and improve adherence. Strengthening and/or increasing human resources and improving work conditions may help improve the TB control program. Because of the economic difficulties and traffic problems patients experience in these remote and poor areas of China, providing additional incentives to support patients like reimbursement of costs related to TB treatment like for transport may also help improve adherence to both medical check-ups and drug treatment.

There are three main limitations to this study. Firstly, 6% of sampled patients did not participate. It is not clear to what extent they are different from participants and thus to what extent this may have caused bias. However, as the non-participation rate was low, we do not expect that it biased our results considerably. Secondly, as patients were interviewed after treatment, they may not have remembered all details correctly. This may have made results more imprecise. It also is possible that patients were reluctant to admit to missing doses of drugs, despite the explanation that the answers would have no consequences for the patients whatsoever. Thirdly, we were not able to include a qualitative study component with in depth interviews in order to obtain insight into reasons for non-DOT and non-compliance.

## Conclusions

In these mountainous areas of China, the TB control program is not fully functioning according to the guidelines. The majority of patients are not treated under direct observation, while direct observation by health care staff was associated with better adherence, both to drug therapy and re-examinations. Measures should be taken urgently in these areas to strengthen implementation of the Stop TB strategy.

## Competing interests

The authors declare that they have no competing interests.

## Authors' contributions

SY, WH, SH, SMY, XW, WC and XF all helped to design the study, coordinate data collection, assist in data-analysis, and drafting of the manuscript. HP was involved in coordination and critical reviewing of the manuscript. All authors read and approved the final manuscript.

## Pre-publication history

The pre-publication history for this paper can be accessed here:

http://www.biomedcentral.com/1472-6963/11/341/prepub
